# Feathers as a Tool to Assess Mercury Contamination in Gentoo Penguins: Variations at the Individual Level

**DOI:** 10.1371/journal.pone.0137622

**Published:** 2015-09-09

**Authors:** Sara Pedro, José C. Xavier, Sílvia Tavares, Phil N. Trathan, Norman Ratcliffe, Vitor H. Paiva, Renata Medeiros, Eduarda Pereira, Miguel A. Pardal

**Affiliations:** 1 Centre of Functional Ecology, Department of Life Sciences, University of Coimbra, Coimbra, Portugal; 2 MARE–Marine and Environmental Sciences Centre, Department of Life Sciences, University of Coimbra, Coimbra, Portugal; 3 British Antarctic Survey, Cambridge, United Kingdom; 4 Cardiff School of Biosciences, Cardiff University, Cardiff, South Glamorgan, Wales, United Kingdom; 5 Centre for Environmental and Marine Studies, Department of Chemistry, University of Aveiro, Aveiro, Portugal; Norwegian Polar Institute, NORWAY

## Abstract

Feathers have been widely used to assess mercury contamination in birds as they reflect metal concentrations accumulated between successive moult periods: they are also easy to sample and have minimum impact on the study birds. Moult is considered the major pathway for mercury excretion in seabirds. Penguins are widely believed to undergo a complete, annual moult during which they do not feed. As penguins lose all their feathers, they are expected to have a low individual-variability in feather mercury concentration as all feathers are formed simultaneously from the same somatic reserves. This assumption is central to penguin studies that use feathers to examine the annual or among-individual variation in mercury concentrations in penguins. To test this assumption, we measured the mercury concentrations in 3–5 body feathers of 52 gentoo penguins (*Pygoscelis papua*) breeding at Bird Island, South Georgia (54°S 38°W). Twenty-five percent of the penguins studied showed substantial within-individual variation in the amount of mercury in their feathers (Coefficient of Variation: 34.7–96.7%). This variation may be caused by differences in moult patterns among individuals within the population leading to different interpretations in the overall population. Further investigation is now needed to fully understand individual variation in penguins’ moult.

## Introduction

The increasing concentration of contaminants in the environment and the need to understand their effects on wildlife are some of the main reasons for the development of bio monitoring programs [1]. Seabirds are often used as bio monitors because of their ease of access for study, their role in the ecosystem as top predators, and the different ranges over which they forage [2,3]. Many seabird species are now considered at risk and in need of protection as they face many and diverse threats in their natural habitat [4]. This fact reinforces the need to collect more information and to use non-invasive methods in defined monitoring programs [5]. Given this perspective, feathers are now often used for measuring various parameters as they can be collected with minimum harm to birds (e.g. feathers can be collected from the ground in penguin colonies, or quickly from live individuals [2]). Feathers can also be stored for years and do not require refrigeration, this therefore allows long-term studies to be conducted (i.e. sampling feather from museums specimens) [3,6,7]. Various contaminants circulating in blood can be sequestered in feathers during their formation, where the contaminants remain physically stable [8]. Mercury is one such contaminant which has been associated with adverse effects on seabird reproductive output, behavior and survival, which has led to it becoming a focus of pollutant monitoring programs [9]. Unlike other contaminants, mercury concentrations in feathers has a much greater internal than external origin, reflecting the mercury dietary intake of mercury prior to feather growth [10–13].

The timing and sequence of moult are factors that are related to a seabird species’ lifecycle, so it is important to take any differences into account which can influence mercury concentrations between species (e.g. [14]), individuals (e.g. [15,16]) and even between feathers from the same individual (e.g. [17]). For example, barn owls (*Tyto alba*) have an irregular and incomplete moult [18], while bald eagles (*Haliaeetus leucocephalus*) may take more than one moult to replace all their primary feathers [19]. In fact, large seabirds, such as albatrosses, do not replace all their flight feathers in one single moult (due to time constraints and reproduction costs); in some cases they take three moulting periods to complete the process [20–22]. Body feathers have been reported to show significant variation in mercury concentrations [16,23]. A high level of variability between body feathers from the same individuals has been reported in Arctic terns (*Sterna paradisaea*), common terns (*Sterna hirundo*) and Leach’s storm-petrels (*Oceanodroma leucorhoa*), which has been related to a long moulting period that occurs during migration to different regions where mercury contamination concentrations may differ [14]. Therefore, the use of more than one feather has been recommended when assessing mercury loads in such species, in order to average out within-individual variation in mercury concentrations. Recently, the use of birds with a synchronous moult, such as penguins and seabird fledglings, has been recommended as suitable for monitoring contaminants in seabird communities [17]. This is because of their synchronous moult that can be defined as a moult in which all body feathers grow simultaneously, at a constant rate and in a short period of time.

Unlike other seabird species, the moult in penguins has a simple pattern, as they replace all their feathers over a period of two to five weeks [24–27]. During this period they remain ashore and do not feed. Therefore, it is anticipated that with this moulting pattern all body feathers should show similar burdens of mercury within individuals as the feathers were formed simultaneously from the same somatic resources [27]. In this study we set out to test this hypothesis using gentoo penguins, that is that variation in individual moulting patterns may occur. In this context, our aims for this study were 1) to assess if there was within-individual variability in the mercury content of body feathers from gentoo penguins; 2) to evaluate the causes of such variability given the assumed synchronous moulting pattern; and 3) to discuss future studies that should be performed to clarify the occurrence of variation in penguins’ moult.

## Material and Methods

### Ethics statement

This project was approved by the Animal Ethics Committee of British Antarctic Survey (BAS) and under a permit issued by the Government of South Georgia. There was no evidence of any prejudicial effects during fieldwork procedures, either on gentoo penguins behavior, breeding success or survival.

### Fieldwork

Sampling procedures and analyses have been previously described [28]. Samples were collected between June and September of 2009 from gentoo penguins breeding at Bird Island, South Georgia (54°S 38°W) after their moult (gentoo penguins moult between March and April [24]). Penguins were randomly selected when returning from sea and coming out of the water at dusk. penguin was handled as follows: after putting a cover on the penguins head (to reduce visual stimuli for the bird), five to seven chest feathers were collected. Feathers were then stored in polyethylene bags until analysis. It should be noted that sampled feathers were cleaned but not washed with organic solvents prior to mercury quantification. The solvents are used to remove any possible surface contamination. By not washing the feathers during their preparation for mercury analysis it is plausible that this could have introduced some bias for the mercury determination [29,30]. However, this should not influence the overall interpretation of the results, since all feathers were processed in exactly the same way. Also, only the tips of the feathers are generally exposed to exogenous mercury, since they overlap one another [30]. Total mercury analyses were performed by thermal decomposition atomic absorption spectrometry with gold amalgamation using a LECO AMA-254. The analysis of certified reference material was performed to guarantee accuracy and precision of the method (IAEA-407 fish tissue certified value = 0.220 ± 0.006 mg kg^-1^ dry mass, recovery of 101.5% ± 6.25%, n = 55). Three feathers were analysed for each penguin. When these feathers showed high variation in the mercury concentration values (SD ≥ 42%, see results in S1 Table), two additional feathers (five in total) were analysed. Every day, mercury concentrations were corrected according to the certified reference material. Blanks were analysed at the beginning and between different feather samples. Based on the blanks, our limit of detection was 0.0638 ng and the mean concentrations for blanks ranged from 0.0035 to 0.0589 ng.

### Data analysis

Linear mixed-effect models (LMMs) were used to test the repeatability of feather mercury concentrations within all individuals. The variance explained by the model (*d*; the between-individual variance), and the residual variance (σ) were used to calculate the intra-class correlation coefficient (ICC) following the formula *d* ^2^/(*d* ^2^ + *σ* ^2^), as a measure of repeatability [17,31]. ICC varies between 0 and 1. ICC values close to one mean that differences between individuals explain most of the variance. Significant differences were considered when p<0.05. Reported values are mean ± SD, unless otherwise stated.

## Results

**Fig 1:**
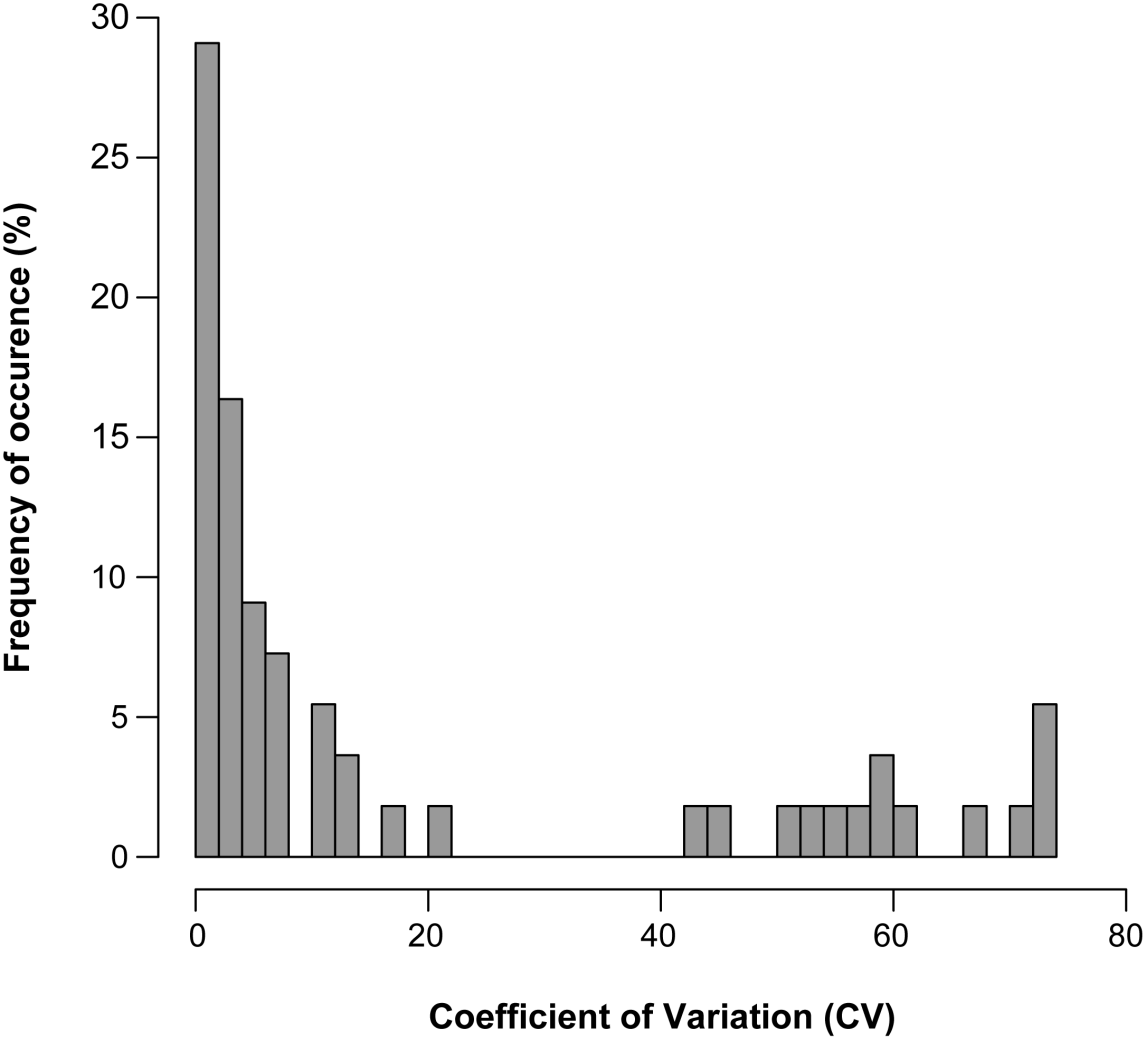
Frequency of occurrence for the Coefficient of Variation (CV) of the mercury content values. Three body feathers from 55 gentoo penguins analyzed.

Mercury concentrations in the population ranged from 0.15–3.10 mg kg^-1^. The coefficient of variation (CV) of the overall sampled population of gentoo penguins varied between 0.23–73.17% (Table 1) and showed a clear bimodal distribution (Fig 1). Birds 42–55 exhibited a very high CV value (Table 1), even when determining the mercury content of more than three body feathers (S1 Table).

**Table 1:** Mercury concentration in gentoo penguins’ body feathers (mg Kg^-1^).

Bird	SD	Minimum	Maximum	CV
1	0.01	0.80	0.81	0.23
2	0.01	0.67	0.68	0.25
3	0.01	1.23	1.24	0.33
4	0.01	1.38	1.39	0.34
5	0.01	1.48	1.50	0.53
6	0.01	0.82	0.84	0.66
7	0.01	0.88	0.89	0.68
8	0.01	0.65	0.66	0.71
9	0.03	2.49	2.54	1.07
10	0.02	1.41	1.44	1.10
11	0.03	2.62	2.68	1.11
12	0.02	1.47	1.51	1.20
13	0.01	0.66	0.69	1.70
14	0.02	0.95	0.99	1.70
15	0.01	0.27	0.28	1.93
16	0.01	0.75	0.78	1.94
17	0.02	1.02	1.06	2.06
18	0.02	0.75	0.78	2.10
19	0.07	3.02	3.15	2.16
20	0.01	0.51	0.53	2.17
21	0.04	1.64	1.71	2.31
22	0.01	0.20	0.21	2.54
23	0.03	1.07	1.13	2.58
24	0.05	1.30	1.39	3.65
25	0.01	0.31	0.33	3.83
26	0.03	0.59	0.64	4.47
27	0.01	0.24	0.26	4.52
28	0.14	2.79	3.04	4.62
29	0.01	0.14	0.15	4.71
30	0.01	0.19	0.21	5.45
31	0.01	0.18	0.20	6.04
32	0.06	0.85	0.97	6.72
33	0.01	0.19	0.22	6.92
34	0.03	0.39	0.44	7.20
35	0.04	0.37	0.45	10.34
36	0.18	1.45	1.79	11.02
37	0.05	0.34	0.43	11.85
38	0.03	0.21	0.27	13.03
39	0.14	0.90	1.18	13.54
40	0.05	0.25	0.34	17.10
41	0.25	0.91	1.39	21.14
**42**	**0.48**	**0.55**	**1.38**	**43.62**
**43**	**0.29**	**0.30**	**0.81**	**45.86**
**44**	**0.22**	**0.30**	**0.68**	**50.90**
**45**	**0.15**	**0.20**	**0.46**	**52.93**
**46**	**0.61**	**0.43**	**1.52**	**54.02**
**47**	**0.43**	**0.26**	**1.01**	**56.36**
**48**	**0.80**	**0.43**	**1.93**	**59.88**
**49**	**0.82**	**0.42**	**1.87**	**59.92**
**50**	**0.63**	**0.63**	**1.75**	**61.51**
**51**	**0.46**	**0.43**	**1.23**	**66.27**
**52**	**0.55**	**0.15**	**1.11**	**70.53**
**53**	**1.33**	**0.31**	**2.64**	**72.12**
**54**	**0.69**	**0.53**	**1.75**	**72.80**
**55**	**0.38**	**0.29**	**0.96**	**73.17**

Values are means of 3 feathers per individual bird. SD—Standard Deviation. CV—Coefficient of Variation (%). Bold area—birds with high CV

The overall population of gentoo penguins showed a low intra class correlation coefficient (ICC) value. The analysis of further feathers (N = 5) from the individuals with high CV did not change the high CV pattern, with values ranging between (34.68–96.65%; S1 Table).

## Discussion

Previously, low within-individual variability has been reported in analyses of king penguins (*Aptenodytes patagonicus*) feathers, such that the authors recommended the use of seabirds with a synchronous moulting pattern, such as penguins, in Southern Ocean monitoring programs [17]. In addition, penguins were considered to be a more precise indicator of mercury concentrations in the marine ecosystem than seabirds that varied their foraging ranges and diet during moult [27]. However, our results suggest that greater caution may be needed as our study demonstrates that high within-individual variability in mercury concentrations occurs in penguin body feathers. Variation in mercury concentrations does occur at the individual level, at least in gentoo penguins, which may then influence levels of perceived accuracy in using penguin feathers to monitor trends in mercury pollution.

The high coefficient of variation and the difference between the maximum and minimum mercury concentrations in the feathers of gentoo penguins (Table 1), suggest a variable pattern of moult among individuals in our study species. Significant differences between feathers at the individual level have been reported previously for different penguin species [27]. Nevertheless, the CV in that study was not as high as we report in this study. Such variability in the mercury content of body feathers of gentoo penguins might be explained by more than one stage of feather formation within a single moult. During moult mercury concentrations decrease gradually in blood while this contaminant is allocated to growing feathers [32]. As a consequence, body feathers grown earlier will display higher mercury concentrations than those grown later, as suggested by previous studies [14,32]. However it does not seem plausible that this pattern of gradual reduction in blood mercury concentrations could explain the high within-individual variability in feather mercury concentrations, due to the short period of time they take to complete moult compared to flying birds. Other possible reasons might include deposition of mercury in feathers from external origins [32]. Since the feathers were not washed in our study, external mercury deposited on the surface of the sampled feathers could have added some unquantifiable error in our mercury results. Nevertheless, contamination by external origins during moult should be very low compared with mercury integrated in feathers from internal sources [10–12]. In addition, as all individual feathers went through a similar treatment, any superficial mercury deposition during handling would be standardized across all feathers. An incomplete moult therefore seems to be the most likely explanation for these results, as this variability in feathers may represent lagged periods of environmental mercury availability [20,21,32].

Anecdotal evidence for this comes from a study of chinstrap penguins (*Pygoscelis antarctica*) in which birds were marked with leg rings and dye marks on the chest feathers prior to moult; the marks were still evident the following year after moult in some, but not all, individuals (N. Ratcliffe, pers. obs). An exactly analogous situation has also been observed for king penguins (P. Trathan pers. obs). In combination with our findings, these observations suggest that in some penguin species moult may be complete for some individuals.

In conclusion, incomplete moult would cause uncertainty when analyzing mercury concentrations (or indeed other stable components within the feather), which would reduce the temporal accuracy of annual monitoring programs. In addition, it may be a confounding factor when examining sources of variation in mercury concentrations among individuals (e.g. differences in diet, sex and age [31]), as some of the variation may be due to mercury concentrations available in different years. Recent studies [17,27] considered that when using penguins as bio monitors any quantity of body feathers would give the same information on mercury burdens. However, results in this study show potential variation in moult patterns among individual gentoo penguins that may give rise to uncertainties about the best method to use when evaluating the metal content from feathers. Furthermore, an incomplete moult may also affect the results of dietary studies using inter-annual variation on stable isotopes in feathers. Further empirical investigation is required to test the assumption of complete annual moult in penguin species, using for example, dye experiments, on species with existing evidence of an incomplete moult (N. Ratcliffe, pers. Obs; P. Trathan pers. obs).

## Supporting Information

S1 Table Mercury concentration (mg Kg^-1^) in gentoo penguins’ body feathers of individuals with a Coefficient of Variation (CV) ≥ 42%. (DOCX)
