# Supplementary material for: Feathers as a Tool to Assess Mercury Contamination in Gentoo Penguins: Variations at the Individual Level
Source: PLoS One. 2015 Sep 9;10(9):e0137622. doi: 10.1371/journal.pone.0137622 (PMC4564222; doi:10.1371/journal.pone.0137622)
Supplement: S1 Table — (DOCX) [file pone.0137622.s001.docx]

S1 Table. Mercury concentration (mg Kg^-1^) in gentoo penguins’ body feathers of individuals with a Coefficient of Variation (CV) ≥ 42%.

| **Bird** | **Sex** | **Mass** | **Mean** | **SD** | **Min** | **Max** | **CV** |
| --- | --- | --- | --- | --- | --- | --- | --- |
| 42 | M | 5050 | 1.16 | 0.42 | 0.55 | 1.44 | 36.10 |
| 43 | F | 5480 | 0.49 | 0.27 | 0.25 | 0.81 | 55.60 |
| 44 | F | 4780 | 0.55 | 0.19 | 0.30 | 0.68 | 34.68 |
| 45 | F | 5450 | 0.33 | 0.14 | 0.20 | 0.46 | 42.47 |
| 46 | F | 5130 | 1.13 | 0.56 | 0.39 | 1.52 | 49.69 |
| 47 | M | 6730 | 0.79 | 0.36 | 0.26 | 1.02 | 45.66 |
| 48 | M | 6300 | 1.03 | 0.72 | 0.43 | 1.93 | 69.62 |
| 49 | M | 7350 | 1.13 | 0.76 | 0.42 | 1.87 | 67.75 |
| 50 | M | 6130 | 1.20 | 0.69 | 0.63 | 2.18 | 57.10 |
| 51 | F | 6380 | 0.69 | 0.40 | 0.42 | 1.23 | 58.30 |
| 52 | M | 6150 | 0.68 | 0.51 | 0.14 | 1.11 | 74.06 |
| 53 | M | 6900 | 1.31 | 1.27 | 0.29 | 2.78 | 96.65 |
| 54 | M | 6950 | 0.95 | 0.76 | 0.15 | 1.75 | 80.35 |
| 55 | F | 5250 | 0.66 | 0.34 | 0.29 | 0.96 | 51.32 |

Values are means of 5 feathers per individual bird. SD – Standard Deviation. CV – Coefficient of Variation (%)
